# Bridging the trust divide: identifying and addressing critical gaps in patient-provider communication

**DOI:** 10.3389/fpsyg.2025.1607653

**Published:** 2026-01-07

**Authors:** Gillie Gabay

**Affiliations:** Achva Academic College, Arugot, Israel

**Keywords:** health psychology and medicine, health promotion, expectations, patient-provider relationship, wellbeing, patient trust, patient-provider communication

## 1 Introduction

Patient-provider encounters are the cornerstone of Medicine, particularly with hospitalized patients (Schattner, 2025). Patient-provider interactions strongly influence patient trust, which is crucial for medication adherence, self-management of illness, fewer readmissions, improved long-term health, wellbeing, and quality of life (Gabay, 2015, 2019, 2023; Hassa et al., 2025). Patient trust is defined as the acceptance of a vulnerable situation in which one party believes that “at the moment of truth,” the other party will take care of its interests (Gabay, 2019; Rousseau et al., 1998). There is an agreement regarding the conceptualization of patient trust in providers, its importance, antecedents, and outcomes (Hassa et al., 2025; Dinç and Gastmans, 2013).

### 1.1 Antecedents of trust

Patient satisfaction played a partial mediating role between perceived physician empathy and patient trust (Bai et al., 2025). Moderate to strong correlations between physician empathy, and patient overall trust, and patient trust in physician's benevolence and competence, and the physician-patient relationship (Wu et al., 2022). Perceived provider's empathy, and benevolence influenced the patient's evaluation of the provider-patient relationship, directly and indirectly via patient overall trust in the provider underlining the importance of patient belief in physician benevolence and empathy in building trust (Wu et al., 2022). Trust is enhanced by more interaction in which providers pay attention to the real needs and expectations of patients, actively allocate resources to meet these needs, enhance patient satisfaction and establish a harmonious relationship (Yufei and Yidi, 2025). Other key factors influencing trust in providers include competency (technical skills, listening, reliability, honesty, and concern for patient wellbeing), compassion, and effective communication (Blendon et al., 2014; Piette et al., 2005; Boulware et al., 2003; Freburger et al., 2003; Tarrant et al., 2003; Gabay, 2025a; Krupat et al., 2004; Street et al., 2008; Cunningham et al., 2007; Stokes et al., 2005; Tarrant et al., 2010; Franks et al., 2006; Fiscella et al., 2004; Tarrant et al., 2008; Fletcher et al., 2007; Brennan et al., 2006; Epstein et al., 2007; Shenolikar et al., 2004; Epstein et al., 2005; Hillen et al., 2013; Dalton et al., 2014; Shaw and Baker, 2004; Thom et al., 2004; Keating et al., 2004; Keitz et al., 2007; Katon et al., 2010; Hofer et al., 2004; Peck et al., 2004; Thom et al., 2002). Patients also trust providers with strong interpersonal skills and high reputations. Trust increases with the severity or duration of illness, the length of the patient-provider relationship, patient's age, religiosity and education level. Similarity between patient and provider demographics also boosts trust. Meeting patient expectations enhances both satisfaction and trust (Ganesan et al., 2010; Schneider et al., 2004; Rainie, 2009; Brown and Bussell, 2011). A trend analysis indicated that although shared decision-making and patient empowerment enhance patient trust in the provider, significant barriers remain (Song et al., 2025).

### 1.2 Barriers to trust

Unmet patient expectations from the provider and unmet provider expectations from patients, in medical encounters, can hinder trust, even when the delivered care is optimal. Mutual awareness of gaps in unmet expectations can avoid the breach of patient trust which has been declining across 29 countries (Rousseau et al., 1998). Poor bedside manners or cultural incompetence can erode trust (Franks et al., 2006; Fiscella et al., 2004; Tarrant et al., 2008). Efforts to enhance trust through improving patient experiences are ongoing but the greatest obstacle to trust is the fundamental mismatch between the providers' concerns in the patient-provider encounter, and the patients' concerns in the encounter (Schattner, 2025). Due to their immediate clinical priorities' providers are task-oriented while patients are concerned about humanistic and relational needs (Schattner, 2025). Also, providers are mainly occupied with the burden of overcoming a set of complex cognitive tasks over a given, relatively short time, assembling and analyzing the patient's intricate history, examination, and test results, battling information overload to identify the most critical, current problem and its management (Gabay and Ben-Asher, 2022). This paper highlights gaps in orientations and expectations between patients and providers, affecting patient trust, especially in the absence of clear communication.

## 2 Methods

### 2.1 Selection criteria, recruitment, and sample of patients

A maximum variation approach was employed to recruit patients, enabling the inclusion of a wide range of perspectives (Denzin and Lincoln, 2011). Participants were 12 secular Israelis (six men and six women), ages 29–81, with diversity in participants' age, gender, geography, illnesses, profession, and work status. Ten were married, one single, and one divorced. Participants were hospitalized in a large hospital (1,202–3,200 beds) or medium hospital (300–700 beds). Narratives provided adequate information power (Malterud et al., 2016). Participants were hospitalized due to cancer, heart disease, neurological disorders, or accidents that put them in mortal danger. A snowball sampling was used to locate subjects in their initial recovery process upon discharge from acute-care setting in a public general hospital. While the average hospitalization lasts 3–4 days, participants were hospitalized for about 3 weeks. Interviews were audio-taped, transcribed verbatim, and translated into English. Table 1 presents sample demographics.

**Table 1:** Sample demographics.

**Age group**	**Disease**	**Profession and status**	**No. of children**
Young (29–36); four participants	Spine cancer; uterine cancer; a neurological disorder; arms and palms crushed	Software developer (employed); engineer (self-employed); dancer (self-employed); designer (self-employed)	0–2
Middle (42–58); four participants	Breast cancer; an upper limb injury; lung cancer; a neurological disorder	Teacher (employed); Software engineer (employed); architect (self-employed); photographer (employed)	2–4
Elders (66–81); six participants	Stemum cancer; neurological; uterus cancer; heart failure (2); and allergies	Consultant (self-employed); insurance agent (employed); psychotherapist (self-employed); Social psychologist (Retired) and a lecturer (employed)	2–4

### 2.2 Selection criteria and recruitment of providers

Following hospital approvals, this author presented the study in staff meetings. Providers interested in participating were invited to contact the author. Most providers contacted the author who assured them anonymous and confidential participation concealing any information that could identify them or the hospitals (Morse and Coulehan, 2015). Interviews lasted between 90 and 120 min were recorded and transcribed verbatim.

### 2.3 Data collection of patient and provider data

Narrative interviews, an effective method for exploring the lived experiences of patients and providers, assigning meaning to them, and elucidating subjective truths, were performed (Josselson, 2013). Typical of narrative interviews, the author asked only one general open-ended question inviting interviewees to share the narrative they choose to share with no predetermined directions (Josselson, 2013). The question for providers was: “What has been your experience since the beginning of your work here?”

Patient were asked: “Please tell me, how did you arrive at the hospital and what did you experience there?” From then on, participants, who vividly remembered their hospitalization, shared their experience from the first appearance of symptoms until discharge. All participants, both patients and providers, were engaged and very emotional as they reflected on how dramatic events impacted them.

### 2.4 Data analysis

The first three steps of the analysis are drawn on the method of selection mechanisms (Gabay, 2015). The fourth step of the analysis draws on the Bricolage approach (Kincheloe, 2001, 2005a,b) The selection mechanisms method aims at a tight correspondence between narrative epistemology and methodology by interpreting narratives in a way that reveals the hospitalization or occupational narrative which each interviewee claimed via the narrative (Gabay, 2015). Each narrative consists of six selection mechanisms, through which biographical facts are chosen, filtered, and sorted to organize events and confirm an endpoint of the narrative.

The following selection mechanisms were identified: inclusion—relating to facts and experiences reported and a common motive among them (e.g., detailing everything that had happened from admission until discharge); sharpening—relating to events that participants highlighted (e.g., lack of privacy as the nurse yelled to other nurses, “Bring me a bedpan”); omission—relating to events that participants viewed as irrelevant to the desired endpoint (e.g., disregarding the dynamics with one's significant other); silencing—relating to events that participants perceived as conflicting with the desired endpoint (e.g., stories of other patients with whom a participant had interaction); flattening—relating to the minimization of events that participants perceived as unimportant (e.g., a participant's distress as a young father who has not seen his children for about a month); and attribution of appropriate meaning—relating to meaning attributed to events that participants found to accord with the endpoints, although they may not necessarily fit their original meaning (e.g., attributing meaning to the degrading attitude of the staff).

In the *third step*, the endpoint of each interview was identified, as it emerged from the analysis using selection mechanisms (e.g., “Although I am dependent on the provider and although I am seen as part of a collective of patients rather than as an individual, I will represent my-self by not signing the informed consent form”). In the *fourth* and final step, common themes in narratives of participants by subgroups, were analyzed, focusing on communication and stressing the impact of communication on patient trust and self-efficacy of providers throughout the trajectory of establishing trust in acute care.

In the fourth step, the Bricolage approach was adopted to examine phenomena from multiple perspectives enhancing rigor, richness, and depth of making meaning in a single study (Denzin and Lincoln, 2011; Kellner, 2009). Kincheloe's Bricolage approach (Kincheloe, 2001, 2005a,b) embraces complexity by viewing subjects not only as detached-in-themselves, but also as connected between them, exploring the role of power in shaping narratives. Adopting Kincheloe's approach, I analyzed commonalities in narratives of subgroups of participants, which entailed dimensions of power relating to interactions with physicians. In this step, the group perspective was added to the analysis, to identify gaps in communication as perceived by providers and patients.

## 3 Results: gaps in patient-provider communication that impede trust

Gaps were identified and analyzed through narrative interviews with patients after lengthy hospitalizations and with providers in Israeli public hospitals (Gabay, 2015, 2019, 2023; Hassa et al., 2025). The gaps in expectations arise from discrepancies between patient expectations and actual experiences, which, without open communication, can erode trust in the provider. Next, is a brief presentation of these gaps and supporting quotes.

### 3.1 Gap 1: patient expectations vs. provider expectations

A chronically ill patient, despite lacking medical training, expects the provider to recognize her experience and expedite processes at the emergency department.

“*This is my fourth hospitalization this year, instead of referring me straight to the ward, I have to wait for hours until the completion of the process in the emergency department, every time, it is exhausting.”*

The provider, however, must assess risks and cannot shorten procedures, which can frustrate the patient and decrease cooperation. Without explaining this, trust may be breached (Gabay, 2019).

### 3.2 Gap 2: provider knowledge vs. patient authority

Some patients, despite their limited medical knowledge, assert their right to make treatment decisions. Providers may feel compelled to intervene, thinking they know best, creating a conflict between patient autonomy and medical authority.

“*What physicians say is not the Bible... they are shooting in the dark. Their knowledge is limited. Every physician has his or her own method, but we know our bodies. We read about the disease, and we know what's right for us.”*

Clear communication is needed to navigate this tension and maintain trust (Liang et al., 2013; Gabay, 2021, 2020).

### 3.3 Gap 3: patient expectation of empathy vs. provider expectation of professionalism

Patients expect empathy, but providers often face burnout and stress, and may struggle to express it. While empathy is essential for patient care, it's not always feasible in a demanding medical environment. Patients may interpret a lack of empathy as unprofessional, reducing trust, even though communication and professionalism can be sufficient for effective care (Gabay and Bokek-Cohen, 2019, 2020).

“*The anesthesiologist was digging to find a vein and connect the tube, it was so painful, I was screaming my lungs out, but he kept going and said nothing. At some point, my surgeon stepped in, heard me and scolded at him to immediately stop: ‘Why don't you do that when she is sedated?!”'*

### 3.4 Gap 4: good communication vs. patient loneliness

“*You lose yourself from day one. It hurts so much. My spirit is broken.... [Quiet]. but I chose to alienate myself... I don't know why... I need it... maybe it's because when I shut my world from the outside, I don't feel like a cripple... I don't know... I have a concealed hate toward anyone who can move their legs, and no one can understand that, but the doctors kept encouraging me, once I was out of the ICU, to invite my friends to visit but I was not ready to talk about books and movies as if everything is the same.”*

A patient's self-alienation and loneliness, driven by pain and fear of the future, may persist despite good communication from the provider. The provider cannot fully understand the patient's pain but can offer compassionate communication that acknowledges loneliness without pretending to know what the patient feels. This approach helps maintain trust (Gabay, 2019).

### 3.5 Gap 5: personal questions vs. professional boundaries

“The doctor was the only one who cared and treated me as a person. He asked how it happened, asked about my children, what ages they were. He said since your children are so young and you may want to have more children, I recommend surgery to avoid future complications. The next day when he saw me, he said nothing to me.”

Providers asking personal questions may create emotional attachment, but inconsistency in behavior can be perceived as hypocritical. To maintain trust, providers should explain the purpose of personal questions as part of medical decisions, and patients should understand this context (Rainie, 2009).

### 3.6 Gap 6: provider routine vs. patient emergency

Providers, immersed in their daily routines, may seem detached from the patient's crisis, leading to feelings of alienation and anxiety. Providers need to be attuned to the patient's needs, especially during recovery, to ensure the patient feels valued and understood, which promotes trust (Gabay, 2019, 2021).

“*I noticed that they are coming and going and talking about the surgery like a regular person would talk about their flower arrangement. Do they even see me? Do they know it's MY cancer?!”*

### 3.7 Gap 7: timing of informed consent vs. patient wellbeing

*Patients are often asked to sign informed consent forms just before surgery, at a time when they are emotionally overwhelmed, increasing stress*.

“*You are laying there naked and awake feeling that you are losing everything. You are just ashes, you are valueless. In those moments, nothing existed around me. I was alone again in the operating room. There is no past and no future. There is only a great grief of this moment, there is crying, excitement, fear, pressure. On the one hand, I was glad that I wasn't in the operating room but on the other hand I wanted to be a minute after the surgery ended. I was laying there for four hours next to the dining room, smelling the hospital cooking. I was lying in the entrance to the operating room, freezing. One nurse came and gave me a heated blanket. The anesthesiologist and the surgeon explained nothing. Then just before entering the operating room I was asked to sign an informed-consent form”*

Providers should give patients time to process the information, allowing them to make informed decisions without feeling pressured, which would strengthen trust (Ganesan et al., 2010; Brown and Bussell, 2011; Phillips et al., 2013).

### 3.8 Gap 8: professionalism in the age of evolving knowledge

As medical knowledge evolves, providers may struggle to admit they do not have all the answers.

“*The procedure was so traumatic. I was very ill. I weighed 47 Kilos instead of the normal weight of 70 pounds. I was in a bad situation looking for a second opinion. The Dr. gave me intensive steroid therapy. The right side of my body was paralyzed twice due to medications. I went to a neurologist who, instead of saying I don't know, gave me a medication for stroke prevention that should not be given to teens at all, it was negligent to give me a medication for adults. I dropped 15 kilos, I was not in school, I was in bed with zero energy. It made me feel worse; I had blood poisoning. I lost an entire school year.”*

Providers must acknowledge their limits and refer patients to specialists demonstrating professionalism, which builds trust by showing confidence in seeking the best care for the patient (Gabay, 2015, 2021).

### 3.9 Gap 9: general vs. unique empathy

Patients expect empathy, but general empathy from providers, especially in acute settings, can be more appropriate than personalized responses.

**“** *After several hours in acute-care, I needed something for the pain again. The physician said, ‘I understand that you are in great pain. Take your pain to a positive place, think Zen'... So condescending!... I was furious with him.”*

To maintain trust, providers should focus on understanding the patient's situation and offering choices, rather than prescribing one-size-fits-all advice (Hofer et al., 2004).

### 3.10 Gap 10: transparency in communication vs. patient weakness

While transparency is important, speaking over a patient's head, especially when they are emotionally overwhelmed or partially awake, can erode trust.

“*The Neurosurgeon came by to see me but didn't see me at all. He hardly spoke with me. When he turned to me he said: ‘You have excess fluid in your head that pressures your brain and explains your cognitive regression, loss of memory, and walking disorder. We will drill a hole in your head, insert a catheter, pull a tube from the brain down through your stomach and regulate the fluids to decrease the pressure in your brain.' [silence]. I was scared to death. I felt intense cold throughout my body.”*

**Figure 1:**
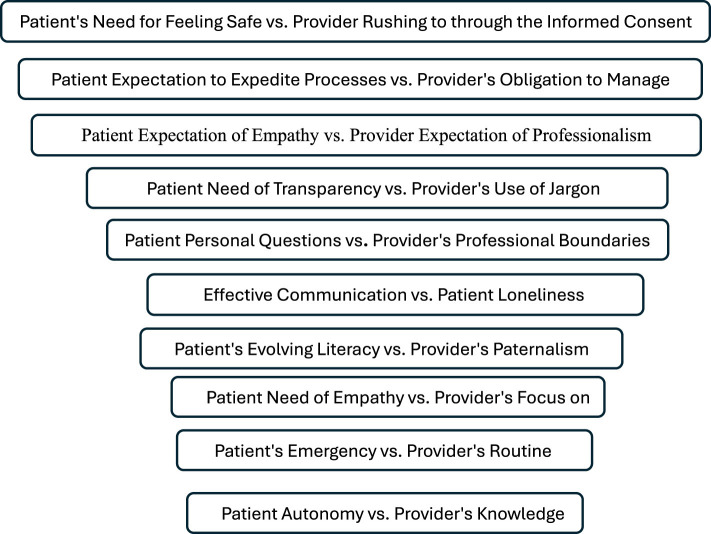
Gaps in patient-provider expectations that impede patient trust.

Providers must be mindful of the patient's emotional state and communicate with sensitivity, avoiding technical jargon and ensuring the patient feels seen and heard (Gabay, 2019, 2021). Figure 1 visualizes the gaps.

## 4 Discussion

This paper emphasizes the importance of effective communication between providers and patients to understand and bridge gaps which are barriers to trust.

In a previous study providers expressed their understanding of the emotional needs of patients, and that while for them working in intensive care is a routine, for patients it is a time of an acute crisis (Gabay, 2025b). Also, providers were aware of the effect of their work stress on them and shared their cognitions, emotions, and behaviors to cope with their work stress (Gabay, 2025b). Due to stress providers reported shifting between a sense of control and lack of control, making them feel helpless, and jeopardizing their emotional wellbeing (Gabay, 2025b). Last, providers attributed their occupational meaningfulness to the difference they make for patients, their communication with patients, and the gratitude from trusting patients which not only deepened their sense of meaningfulness but also, mitigated harsh experiences (Gabay, 2025b).

By encouraging active communication, providers can understand gaps in expectations and improve responsiveness. Providers who understand the gaps in expectations can resolve these issues by choosing alternative solutions (Gabay, 2020; Gabay and Bokek-Cohen, 2019, 2020; Gabay, 2025a). Awareness of these gaps also empowers patients to better understand the complexity of their situation. Managing patient expectations through clear communication can help build and maintain trust. A patient-centered approach should focus on empowering the patient as active players in Managing their health. Collaborative communication should prioritize the patient's actions to improve their illness management, not just the illness itself. Health systems and policymakers should create training programs to enhance providers' awareness of the impact of internal control on patient health promotion and their readiness to adopt collaborative communication skills. Such training will improve patient trust, and the quality of care provided, ultimately benefiting both patients and providers.

### 4.1 Study limitations and directions for future studies

Several limitations of this opinion paper must be acknowledged. First, cultural characteristics of providers and patients as well as the climate at the intensive care unit may have shaped the conclusions regarding communication gaps. Second, it is possible that narratives may evolve past the time of the interviews, affecting the participants' choice of the narratives. Future studies may continue to explore gaps in communication between patients and providers to substantiate the gaps presented herein or reveal additional ones.
